# Genomics of ecological adaptation in Canary Island *Descurainia* (Brassicaceae) and comparisons with other Brassicaceae

**DOI:** 10.1002/ece3.70144

**Published:** 2024-08-08

**Authors:** Amy C. Jackson, Mark A. Carine, Mark A. Chapman

**Affiliations:** ^1^ Biological Sciences University of Southampton Southampton UK; ^2^ Algae, Fungi and Plants Division The Natural History Museum London UK; ^3^ Present address: Royal Botanic Gardens, Kew, Kew Green Richmond Surrey UK

**Keywords:** *Descurainia*, genome, island biogeography, molecular evolution

## Abstract

Oceanic archipelagos provide striking examples of lineages that have radiated over pronounced ecological gradients. Accompanying this diversification, lineages have evolved adaptations allowing survival in extreme environments. Here, we investigate the genomic basis of ecological adaptation in Canary Island *Descurainia* (Brassicaceae), an island relative of *Arabidopsis*. The seven endemic species have diversified in situ along an elevational and ecological gradient, from low‐elevation scrub to high‐elevation sub‐alpine desert. We first generated a reference genome for *Descurainia millefolia,* phylogenetic analysis of which placed it as sister to *D. sophioides*. Ninety‐six gene families were found to be specific to *D. millefolia* and a further 1087 and 1469 gene families have expanded or contracted in size, respectively, along the *D. millefolia* branch. We then employed genome re‐sequencing to sample 14 genomes across the seven species of Canary Island *Descurainia* and an outgroup. Phylogenomic analyses were consistent with previous reconstructions of Canary Island *Descurainia* in resolving low‐ and high‐elevation clades. Using the branch‐site dN/dS method, we detected positive selection for 275 genes on the branch separating the low‐ and high‐elevation species and these positively selected genes (PSGs) were significantly enriched for functions related to reproduction and stress tolerance. Comparing PSGs to those in analyses of adaptation to elevation and/or latitude in other Brassicaceae, we found little evidence of widespread convergence and gene reuse, except for two examples, one of which was a significant overlap between *Descurainia* and *Draba nivalis,* a species restricted to high latitudes. The study of Canary Island *Descurainia* suggests that the transition to high‐elevation environments such as that found in the high mountains of the Canary Islands involves selection on genes related to reproduction and stress tolerance but that repeated evolution across different lineages that have evolved into similar habitats is limited, indicating substantially different molecular trajectories to adaptation.

## Graphical abstract

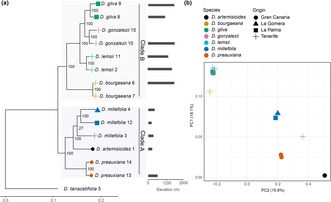

## 1 INTRODUCTION

Islands play a significant role in global biodiversity, hosting 25% of global endemic species richness despite covering less than 7% of the Earth's total land surface area (Paulay, 1994). Endemic lineages on oceanic islands have often adapted and diversified in situ rapidly and across short distances (Emerson, 2002; Warren et al., 2015) and they serve as a model for understanding the drivers of speciation and phenotypic diversification (Burns, 2019). Diversification of oceanic island lineages has involved various processes including isolation and drift following dispersal events between islands, adaptation to a broad spectrum of ecological niches and hybridisation (Fernández‐Palacios et al., 2021; Juan et al., 2000; Losos & Ricklefs, 2009; White et al., 2020). Together, geographical and environmental isolation have served as a powerful framework to understand adaptation and speciation, with implications for biodiversity conservation and climate change resilience (Russell & Kueffer, 2019).

High‐elevation oceanic islands are a particularly useful model due to the large number of endemic species distributed along pronounced environmental and climatic gradients (Halbritter et al., 2018; Steinbauer et al., 2012). High‐elevation habitats on oceanic and continental mountain‐like systems are typically characterised by low temperatures, poor soil, strong winds, frost events and high UV radiation, accompanied by substantial diurnal and annual variation in these parameters (Nagy & Grabherr, 2009). Adaptation to such harsh conditions is often accompanied by drastic morphological and physiological evolution. For example, in the Brassicaceae, high‐ and low‐elevation *Eutrema* species in the Qinghai‐Tibet Plateau are differentiated at genes associated with DNA damage repair, reproduction and cold tolerance (Guo et al., 2018); in *Lepidium meyenii* from the Andes, the selection on genes linked to the development of specialised leaf shapes has been documented (Zhang et al., 2016). Several population genomic analyses of adaptation to elevational differences (e.g. Chapman et al., 2013; Chen et al., 2019; Feng et al., 2022; Li et al., 2023; Ma et al., 2019; Zeng et al., 2020) have identified a range of genes and pathways associated with adaptation along such a gradient, for example, those associated with environmental stressors (e.g. light, temperature and water availability) and reproduction (e.g. vernalisation and flowering time). However, despite their significance, genomic studies focusing on radiations within island systems remain relatively scarce (Cerca et al., 2023). This gap in research limits our understanding of the genetic mechanisms driving adaptation and speciation in these unique ecosystems.

In our study, we focus on Canary Island *Descurainia* Webb & Berthel. (Brassicaceae), an example of a recent plant radiation, with congeners occurring at different elevations (Goodson et al., 2006; Jackson et al., 2023). The Canary Islands is a volcanic oceanic archipelago of eight main islands located ca. 100 km west of North Africa. The archipelago has a rich endemic flora comprising ca. 680 endemic species, constituting 40%–50% of total native flora (Reyes‐Betancort et al., 2008) and provides striking examples of rapid evolutionary radiations (e.g. *Aeonium*, *Argyranthemum* and *Echium*). Endemic species are often restricted to elevational‐environmental belts, which broadly consist of sub‐desert coastal scrub (90–600 m), thermophilous woodlands (300–1100 m), laurel forest (600–1200 m), Canarian pine forest (1100–2100 m) and Dry subalpine zone (2000–3000 m; Fernández‐ Palacios & de Nicolás, 1995).

**FIGURE 1:**
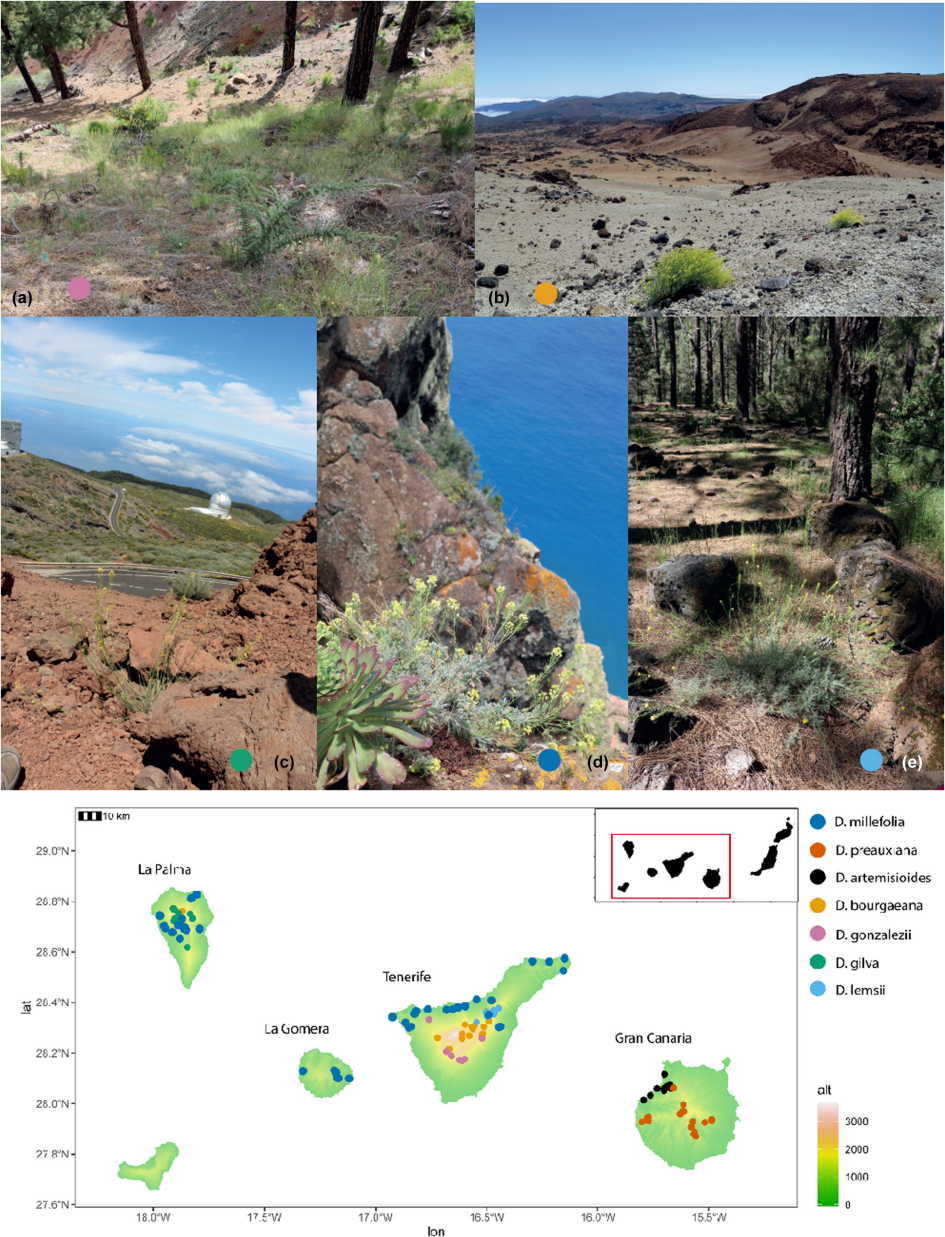
Top: Habitats of *Descurainia* species. (a) *D. gonzalezii* in a pine forest habitat in Tenerife (1750 m), (b) The sub‐ alpine scrub of El Teide, Tenerife (2100 m), with *D. bourgaeana* in the foreground, (c) subalpine scrub of La Palma where *D. gilva* is distributed (2000 m), (d) dry scrublands on Tenerife where *D. millefolia* is often found on the cliffs (250 m), (e) *D. lemsii* distributed on the margins of the pine forest (2000 m). Bottom: Distribution of the seven endemic Canary Island *Descurainia* species across four islands. Each point represents a GPS coordinate (261 individuals) collated from herbarium specimens deposited within the herbarium of the Natural History Museum, London (BM). Terrain colours represent the elevation (m). Photographs were taken by AJ except for panel (b) taken by Rachael Graham (with permission).

*Descurainia* comprises ca. 45 species, distributed across the Americas, Europe and Asia, seven of which are endemic to the Canary Islands where the lineage has diversified within and between four islands (Tenerife, La Palma, La Gomera and Gran Canaria; Figure 1; Goodson et al., 2011). Five species are single island endemics (SIEs): *D. gonzalezii* Svent., and *D. lemsii* Bramwell are endemic to Tenerife, *D. artemisioides* Svent. and *D. preauxiana* (Webb) Webb ex O.E.Schulz to Gran Canaria and *D. gilva* Svent. to La Palma. *D. millefolia* (Jacq.) Webb & Berthel is distributed across three islands and *D. bourgaeana* (E.Fourn.) Webb ex O.E.Schulz across Tenerife with a more recent introduction into La Palma (Jackson et al., 2023). Phylogenetic analysis resolved the seven species into two clades, one distributed largely in hot and dry low‐elevation habitats (<1100 m; *D. millefolia*, *D. artemisioides* and *D. preauxiana*) and the other at cold and wet high‐elevation habitats (typically >1800 m; *D. bourgaeana*, *D. lemsii*, *D. gilva* and *D. gonzalezii*; Jackson et al., 2023). Previous work indicated that climatic differences between these elevations, particularly precipitation and temperature, and strong drivers of the diversification of these two clades (Jackson et al., 2023). Overall, a combination of ecological and geographic processes and hybridisation have contributed to the diversification of this group (Goodson et al., 2006; Jackson et al., 2023).

The subalpine zone of the Canary Islands, where four of the seven species of *Descurainia* occur, is restricted to the high mountains of Tenerife and La Palma. The average annual temperature is low, 6–11°C and diurnal fluctuations high (Bramwell & Caujapé‐Castells, 2011). There is also a frost period which extends from October to May, and snow in the winter (del Arco Aguilar & Rodríguez Delgado, 2018). In contrast to continental mountains, rainfall at the subalpine zone is relatively low (between 350 and 500 mm) compared to mid‐elevation habitats. The harsh abiotic conditions and isolation of the subalpine zones have resulted in a relatively species‐poor flora with high levels of endemism (38%; Irl et al., 2015; Steinbauer et al., 2016).

In this study, we identify the genes that putatively underlie adaptation to high‐altitude habitats within Canary Island *Descurainia* and investigate whether repeated evolution at the gene level has occurred across Brassicaceae by comparison with other species within the family that occur in other high‐elevation or high‐latitude environments. To do this, we (1) generate a reference genome for Canary Island *Descurainia* and resequence an additional 14 genomes representing the seven endemic species, (2) identify signatures of positive selection to reveal genes putatively associated with ecological adaptation in Canary Island *Descurainia* and (3) examine evidence for repeated evolution in the molecular signatures of adaptation to high elevation and high latitude environments across phylogenetically divergent species of the Brassicaceae.

## 2 MATERIALS AND METHODS

### 2.1 *Descurainia millefolia* reference genome sequencing, assembly and annotation

#### 2.1.1 Sample collection and DNA extraction

Leaf tissue was collected from a *D. millefolia* plant cultivated in the School of Biological Sciences, University of Southampton greenhouse facilities grown from seed material obtained from the Universidad Politécnica de Madrid (UPM) seed bank (Madrid, Spain, seed accession number: ETSIA‐246‐1073‐67). The leaves were frozen in liquid nitrogen, and DNA was isolated using a CTAB‐based DNA extraction (Porebski et al., 1997). The extracted DNA was treated with RNase and assessed for molecular weight and concentration. DNA was used to prepare whole‐genome libraries at NovoGene (Hong Kong) for long‐read Pacific Biosciences Sequel sequencing with 1.0 chemistry platform and for short‐read Illumina HiSeq 3000 sequencing (150‐bp paired‐end).

#### 2.1.2 Reference assembly and validation

The draft de novo genome of *D. millefolia* was generated using a hybrid assembly approach that combines short‐ and long‐read sequencing libraries. Hybrid assembly approaches involved supplementing accurate short‐read sequencing data with more error‐prone long‐read sequences to resolve a large and repetitive genome.

The raw data for short‐read sequences were quality checked with FastQC v.3.0 (Andrews, 2010) and adapters were removed using Trimmomatic v.0.32 (Bolger et al., 2014) with the minimum length for a read to be retained at 100 bp. Due to the noise given by the PacBio long‐reads, trimming was not applied. The genome size of *D. millefolia* was estimated from the most optimal k‐mer value for both libraries (short‐ and long‐read) using KmerGenie v.1.7 (Chikhi & Medvedev, 2014).

Two hybrid assembly programmes, namely DBG2OLC (https://github.com/yechengxi/DBG2OLC; Ye et al., 2016) and MaSuRCA v.3.3.0 (Zimin et al., 2013), were selected to ensure the best quality draft assembly possible. The purpose‐built NGS and 3GS hybrid assembler, DBG2OLC, involved three main steps to generate a de novo hybrid assembly. First, de Bruijn graph contigs from the Illumina reads were created using Sparse Assembler (https://github.com/yechengxi/SparseAssembler), with *k* = 119 (based on k‐mer estimation; see Section 3 Results). Second, DBG2OLC mapped these contigs to the long reads to create a backbone assembly (adaptive threshold of 1 × 10^−4^). Finally, a PacBio long‐read aligner, Blasr (github.com/PacificBiosciences/blasr), was used to create a consensus assembly from the previously generated contigs, long reads and the backbone assembly.

MaSuRCA employs a hybrid assembly and has been shown to handle repetitive plant genomes well (Zimin et al., 2017). Here, the k‐mer size was estimated by MaSuRCA from read error rate and coverage, giving a value of 99. Due to the relatively short read length of our PacBio reads (mean 7086 bp; Table S1), three coverage parameters were examined (25, 30 and 35). This assembly was polished using POLCA (Zimin & Salzberg, 2020) to improve consensus accuracy.

Genome quality was assessed using standard assembly statistics generated from QUAST (Gurevich et al., 2013), which includes N50 and contig length. In addition, Benchmarking Universal Single‐Copy Orthologs v.3.0 (BUSCO; Simão et al., 2015) was used to assess genome completeness by identifying single‐copy, duplicated, fragmented or missing BUSCOs. The resulting contigs from the final draft assembly genome were checked for contamination using BLASTn (Altschul et al., 1990) against the NCBI nucleotide database. Identified contaminated contigs were removed using Picard v.2.8.3 (https://broadinstitute.github.io/picard).

The contigs from the final assembly selected were scaffolded with the PacBio long‐reads using SSPACE Long Read v.1.1 (Boetzer & Pirovano, 2014) and gap filled with the Illumina short reads using SSPACE Gap Filler v.1.1. Finally, both sets of trimmed reads were mapped back to the reference genome using BWA‐MEM v.0.7.11 (Li & Durbin, 2009).

#### 2.1.3 Repeat annotation

RepeatModeler v.1.0.11 (Smit & Hubley, 2008) was used for de novo identification of repetitive sequences. These were appended to the *Arabidopsis thaliana* repeat library from RepBase (2017 release), resulting in a final repeat library. This was used to annotate repeats and transposable elements (TEs) in the genome using RepeatMasker v.4.0.9 (Tarailo‐Graovac & Chen, 2009), employing RMBlast v.2.9.0 to search against RepBase (2017 release; Jurka et al., 2005) and Dfam (v.3.0; Storer et al., 2021), using the ‘Viridiplantae’ species function. Protein‐level repeats were identified by searching against the TE protein database using RepeatProteinMask v.4.0.7 (Tarailo‐Graovac & Chen, 2009). Tandem repeats were identified using Tandem Repeat Finder (v.4.09; Benson, 1999).

#### 2.1.4 Structural annotation

Trimmed RNA reads from the close relative *Descurainia bourgaeana* were obtained from White et al. (2016) for initial annotation to train ab initio gene prediction. RNA‐seq reads were mapped to the genome using STAR aligner (Dobin et al., 2013).

De novo gene prediction for the reference genome was carried out with MAKER v.2.31.10 (Cantarel et al., 2008) in conjunction with Augustus v.3.3.3 (Stanke et al., 2006). Training models were obtained from the aligned *D. bourgaeana* RNA‐seq sequence data using the cufflinks protocol (Trapnell et al., 2012). Augustus and SNAP (github.com/KorfLab/SNAP) were used for ab initio gene training using protein datasets from four closely related members of the Brassicaceae, namely *Arabidopsis thaliana* (v. TAIR10; Berardini et al., 2015), *Brassica napus* (Accession: PRJNA546246; Song et al., 2020), *Brassica oleracea* (Accession: GCF_000695525.1; Parkin et al., 2014) and *Capsella rubella* (Accession: GCA_000375325.1; Slotte et al., 2013).

A MAKER‐derived annotation edit distance (AED) score (Eilbeck et al., 2009) was assigned to each gene model. AED scores range between 0 (perfect match to the evidence) and 1 (absence of support). If 90% of predictions display an AED < 0.5, this is considered well‐annotated (Campbell et al., 2014). MAKER was run iteratively three times, providing training files for each run until the AED scores for each gene model plateau.

#### 2.1.5 Functional annotation

To assess putative gene function, MAKER was used to provide functional annotation for genes annotated in the assembly using NCBI BLAST+ and the Uniprot TrEMBl protein dataset (Morgat et al., 2020). Gene ontology (GO) terms were also annotated by searching against UniProt (Viridiplantae). Gene models were used in a BLAST search against the protein dataset using a cut‐off e‐value of 1 × 10^−4^. Since our annotation pipeline uses multiple sources of protein evidence (i.e. RNA‐seq and protein datasets from closely related species) we retained information on the source of evidence used for each protein model.

### 2.2 Comparative phylogenetics

To determine the taxonomic placement of *D. millefolia* within Brassicaceae ‘Lineage I’, we used OrthoFinder v.2.2.7 (Emms & Kelly, 2019) to identify shared orthologues across 13 species from Lineage I, with *Aethionema arabicum* as the outgroup (ry Table S2). Orthofinder was run with the multiple sequence alignment method (−M) and MMseqs2 to cluster proteins by sequence similarity. STAG (Emms & Kelly, 2018) was used to create a consensus tree from aligned concatenated single‐copy orthogroups. STRIDE was used to root the tree (Emms & Kelly, 2017).

Divergence times were estimated from the Orthofinder analysis using MCMCtree in PAML (Phylogenetic Analysis by Maximum Likelihood v.4.8; Yang, 2007). MCMCtree was run using an Approximate Maximum Likelihood method (dos Reis & Yang, 2019), whereby an empirical rate matrix and gamma rates among sites were used to generate a Hessian matrix. A Markov chain Monte Carlo (MCMC) analysis was run for 10,000 generations, with burn‐in of 1000 iterations. Divergence time was calibrated from TimeTree5 (Kumar et al., 2017) for six pairs of genera (Table S3).

To test for significant contractions and expansions of gene families (i.e. orthogroups from OrthoFinder), we used CAFÉ v.5 (https://github.com/hahnlab/CAFE5). The OrthoFinder species tree was transformed into an ultrametric tree using the R packages ‘phytools’ and ‘phangorn’ (Revell, 2012; Schliep, 2011) and used as input. A P value was calculated for each gene family, and we used *p* < .01 to indicate significant gene gains or losses. For each *D. millefolia* gene family significantly expanded or contracted, functional information was inferred based on orthology to *A. thaliana*. KEGG (Kyoto Encyclopaedia of Genes and Genomes) annotations were analysed using agriGO (www.systemsbiology.cau.edu.cn/agriGOv2) with the singular enrichment analysis method and TAIR10 database.

### 2.3 Resequencing and selection analysis

#### 2.3.1 Sample collection, whole‐genome resequencing, mapping, SNP calling and filtering

A total of 14 samples were obtained to represent the seven Canary Island *Descurainia* species and one sample for an outgroup (Table 1). Eleven of the Canary Island samples were taken from leaf material collected in 2019 and preserved in silica gel (permit numbers: Parque Nacional del Teide, no. 42917710D; Cabildo de la Palma, no. 2019004595; Cabildo de la Gomera, no. 9319; Cabildo de Gran Canaria, no. 14328). Herbarium vouchers have been deposited in the Natural History Museum, London (BM). Three additional samples of Canary Island *Descurainia* were obtained from the UPM seedbank. A sample of DNA from the closest continental relative, *D. tanacetifolia*, utilised in the Goodson et al. (2006) study was kindly provided by Robert K. Jansen (The University of Texas at Austin) as the outgroup. All DNA was extracted using a CTAB‐based method (Porebski et al., 1997). Sequencing (150 bp paired‐end) was carried out by Novogene (Hong Kong) on an Illumina HISEQ X 10 Sequencer (Illumina Inc.).

**TABLE 1:** Canary Island *Descurainia* individuals and outgroup selected for whole genome resequencing.

Species	Study ID	Origin	Elevation (m)	Habitat type	Voucher ID
*D. artemisioides*	1	GC	410	Subdesert scrub	BM013825841
*D. bourgaeana*	6	TEN	2058	Subalpine	BM013825795
*D. bourgaeana*	7	TEN	N/A	Subalpine	N/A^b^
*D. gilva*	8	LP	1275	Subalpine	BM013825888
*D. gilva*	9	LP	2073	Subalpine	BM013825861
*D. gonzalezii*	10	TEN	2064	Southern‐slope pine forest and pine forest/subalpine border	BM013825808
*D. gonzalezii*	15	TEN	N/A	Southern‐slope pine forest and pine forest/subalpine border	N/A^b^
*D. lemsii*	2	TEN	1963	Northern‐slope pine forest/subalpine border	BM013825789
*D. lemsii*	11	TEN	1615	Northern‐slope pine forest/subalpine border	BM013825782
*D. millefolia*	3	TEN	540	Subdesert scrub	BM013825821
*D. millefolia*	4	LG	605	Subdesert scrub	BM013825833
*D. millefolia*	12	LP	432	Subdesert scrub	BM013825880
*D. preauxiana*	13	GC	845	Subdesert scrub	BM013825850
*D. preauxiana*	14	GC	N/A	Subdesert scrub	N/A^b^
*D. tanacetifolia* ^a^	5	(Spain)	N/A	(Mediterranean)	N/A

*Note*: Habitat descriptions for Canary Island *Descurainia* are based on Bramwell. (1977) and personal observations (ACJ).

Abbreviations: GC, Gran Canaria, LG, La Gomera, LP, La Palma, TEN, Tenerife.

^a^ DNA obtained from Robert K. Jansen and utilised in Goodson et al. (2006).

^b^ DNA extracted from plants grown from seed supplied by the UPM seed bank.

Read quality was assessed using FASTQC, and reads were trimmed using Trimmomatic (Bolger et al., 2014) using settings LEADING:3 TRAILING:3 SLIDINGWINDOW:4:15 and reads shorter than 65 bp were excluded. Trimmed reads were mapped to the *D. millefolia* annotated reference genome using BWA‐mem (v.0.7.17; Li & Durbin, 2009) with default parameters. Outputted BAM files were sorted, and unmapped reads were discarded using SAMtools v.1.3.1 (Li et al., 2009). PCR duplicates were removed from the mapped reads using Picard (github.com/broadinstitute/picard; accessed 2020). Chloroplast regions were discarded.

BAM files were processed with SAMtools v.1.15 mpileup to call variants and with BCFtools to identify INDELs, and then converted into a VCF file. VCFtools v.0.1.13 (Danecek et al., 2011) was used to filter the VCF file by a minimum and maximum depth of 10 and 50, respectively, and to only include SNPs with a minor allele frequency of >0.1, a quality value >30 and with <50% missing data. Using the GFF annotation (above), the VCF files were annotated as exons, introns, coding and intergenic using SNPeff (Cingolani et al., 2012).

#### 2.3.2 Species relationships

Phylogenetic reconstruction within Canarian *Descurainia* was carried out using filtered SNPs and the Maximum‐Likelihood (ML) method. SNPs were converted into phylip format using BioPython v.1.79, and then RAxML v.8.2.9 (Stamatakis, 2014) was employed with a GTRGAMMA evolutionary substitution model and 2000 partitions. *Descurainia tanacetifolia* was used as the outgroup, and 1000 bootstraps were computed. To examine the clustering of samples, principal component analysis (PCA) was carried out on the filtered VCF file using the pca function in PLINK, and the first two PCAs visualised using the R package ‘ggplot2’ (Wickham, 2009).

Note that we previously identified a hybrid origin for *D. gilva* (Jackson et al., 2023), however, given that 99% of the hybrid genome was shared with one parent (*D. gonzalezii*), we assume that this hybridisation event will have minimal effect on the phylogeny and the identification of positively selected genes.

#### 2.3.3 Identification of genes under selection

To identify candidate genes associated with high‐elevation adaptation in Canary Island *Descurainia*, CODEML within PAML v.4.0 (Yang, 2007) was used to perform a dN/dS (ω) analysis. A Branch‐Site Model was employed to detect positive selection on the branch separating the low‐ and high‐elevation taxa (i.e. the foreground branch; see Section 3 Results).

To obtain coding sequences (CDSs) for the CODEML analysis, we first created a consensus genome for each of the mapped 14 WGRS genomes using our BAM files and the SAMtools consensus function. These were converted to a FASTA file and bases with quality <20 masked to N. CDSs and their translations were extracted for each species (the sample with the greatest mapping percentage was selected; eight in total) using the *D. millefolia* genome GFF and Gffread (Pertea & Pertea, 2020) with the ‐*w* and ‐*y* parameters. Alignments of the CDSs and protein sequences were performed using BWA. PAL2NAL (Suyama et al., 2006) was then used to create codon alignments matching the CDS alignment. The resulting codon alignments were used to generate unrooted gene trees using RAxMLHPC.

CODEML was used to construct (i) a null model (M1) for each gene where all branches were limited to *ω* = 1 (i.e. excluding selection), and (ii) an alternative model (bsA) where the foreground branch was allowed to display *ω* > 1 (i.e. including positive selection). CODEML was first run with a subset of 5000 genes with different ω parameters, and the ω value with the highest average likelihood score (see Section 3 Results) was used in the full analysis. ete‐evol within the python package ‘ETE toolkit’ (Huerta‐Cepas et al., 2016) was used to automate the marking of foreground branches and perform the CODEML branch‐site analysis on each gene tree. Likelihood ratio tests (LRTs) were used to compare the two models (M1 vs. bsA) and adjusted for multiple testing using the Bonferroni correction (Benjamini & Hochberg, 1995).

We performed GO (Ashburner et al., 2000) enrichment analysis under three sub‐ontology categories, biological process (BP), molecular function (MF) and cellular component (CC), to identify enriched GO terms associated with our positively selected genes (PSGs; FDR < 0.05), using the ShinyGO R package (Ge et al., 2020) and FDR cut‐off of 0.05.

### 2.4 Repeated evolution across the Brassicaceae

We reviewed studies that detected genes under selection in other members of the Brassicaceae that showed adaptation to either high latitude or high elevation. Where gene lists were available in Araport format these were downloaded (seven studies; see Section 3 results) and used for cross‐study comparison. To test for a greater‐than‐expected number of overlapping PSGs, we used Fisher's exact tests, calculated using the ‘SuperExactTest’ package in R (Wang et al., 2015) and we calculated the probability of gene reuse between any significant (*p* < .05; see Section 3 Results) overlap. Gene reuse is calculated as the number of parallel candidate items divided by the total number of candidate items between them (i.e. the union of candidate lists from both lineages; Conte et al., 2012). As the ancestral phenotypes of the included species are ambiguous, we refer to significant overlapping genetic patterns as ‘repeated evolution’ (Cerca, 2023).

## 3 RESULTS

### 3.1 *Descurainia millefolia* reference genome sequencing, assembly and annotation

We obtained 111 M Illumina paired‐end short reads (ca. 83 X genome coverage; Table S1). These reads were then quality checked, removing adapters and trimming the first ten bp, retaining 98.86% of reads. PacBio sequencing resulted in 515 k reads (ca. 18 X genome coverage; average subread length 7067 bps; N50 of 1.1 kb; Table S1). The genome size of *D. millefolia* is reported as 0.22 pg/1C (Lysak et al., 2009) or ca. 216 Mb. Using k‐mer counting, we estimated that the genome size of our sample is between 183 Mb (based on Illumina reads) and 223 Mb (PacBio), therefore our assemblies were carried out using an estimate of 200 Mb.

We used two assembly approaches, DBG2OLC and MaSuRCA and outputs were compared for the total assembly length and N50. Based on this, the optimal parameters for DBG2OLC (Table S4) provided a poorer draft genome assembly (smaller overall size and lower N50) than the alternative MaSuRCA (Table 2). For MaSuRCA, all three coverage parameters (25, 30 and 35) resulted in N50 > 200 KB and a total length close to our genome size estimates (Table 2). We selected the MaSuRCA assembly with the greatest N50 (i.e. coverage 30) for annotation and use in the selection analysis.

**TABLE 2:** QUAST summary statistics of the raw genome assemblies generated by DBG2OLC and MaSuRCA assembly methods (before scaffolding and space‐filling) for *Descurainia millefolia*.

QUAST statistic	Assembly			
	DBG2OLC	MaSuRCA – 25 coverage	MaSuRCA – 30 coverage	MaSuRCA – 35 coverage
# contigs	2110	3021	2816	3048
# contigs (≥10,000 bp)	1836	1973	1875	1939
# contigs (≥50,000 bp)	717	716	669	679
Largest contig (bp)	2,366,526	2,539,606	1,957,821	2,138,193
Total length (bp)^a^	155,969,182	176,914,168	174,396,279	176,629,879
GC (%)	35.32	35.83	35.78	35.80
N50 (bp)^a^	192,527	207,120	223,560	219,265
N75 (bp)	71,503	75,249	79,445	75,913
L50	199	193	176	183
L75	545	543	499	517

*Note*: Parameters performed using the DBG2OLC assembler are found in Table S4.

^a^ Parameters used to help identify which assembly was best.

**FIGURE 2:**
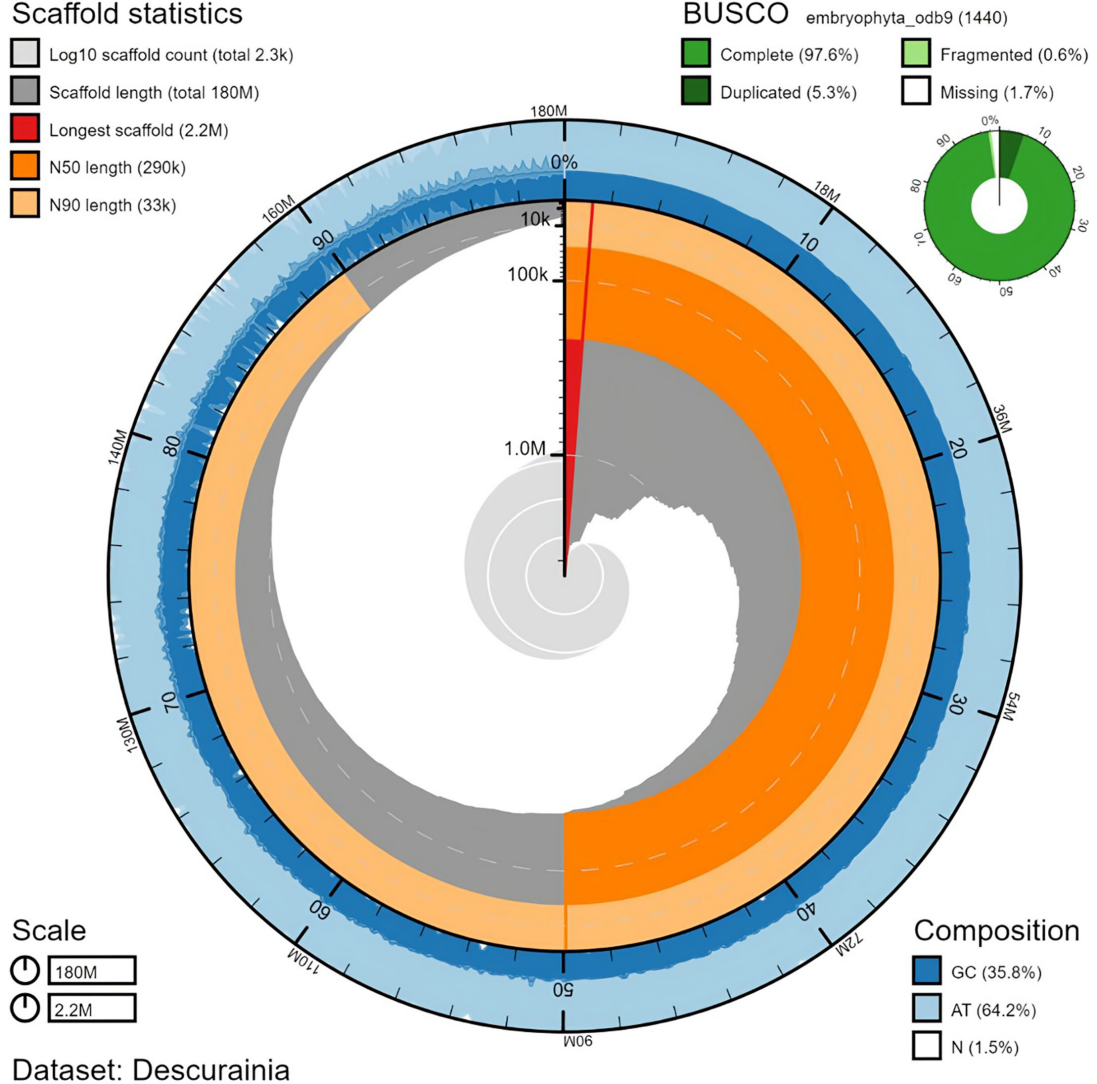
Snail plot summary of the assembly statistics and BUSCO scores for the *Descurainia millefolia* genome. The distribution of record lengths is shown in dark grey in descending order of size, with the plot radius scaled to the longest record present in the assembly (red). Dark and pale orange arcs show the N50 and N90 record lengths respectively. The pale grey spiral shows the cumulative record count on a log scale, with white scale lines showing successive orders of magnitude. The blue and pale‐blue area around the outside of the plot shows the distribution of GC, AT and N percentages in the same bins as the inner plot. BUSCO scores are presented in the top right, using the background genes of embryophyte (V09; 1440). Genome assembly was visualised using BlobTools (Laetsch & Blaxter, 2017). Numbers in the bottom left represent genome size (top) and largest contig (bottom).

Using our long reads, SSPACE scaffolded the assembly from 2816 contigs to 2274 scaffolds and gap closing closed 2.3% of gaps and 6.7% of nucleotides. The N50 of the scaffolded assembly was 290 kb, the longest scaffold was 2.17 MB and the genome size was 180 MB, slightly lower than our estimates (Figure 2; Table 3). Completeness of the genome was assessed using BUSCO and the ‘embryophyta09’ dataset; we identified 1406 (97.6%) of 1440 BUSCOs as being complete and only 25 (1.7%) were missing (Figure 2; Table S5).

**TABLE 3:** – Genome and annotation summary statistics of the final de novo assembly and annotation of *Descurainia millefolia*, using a hybrid assembly (MaSuRCA) approach.

Genome statistics	
# Scaffolds	2274
Total length (bp)	179,686,191
Largest contig (bp)	2,172,884
GC (%)	35.81
N50 (bp)	289,995
L50 (bp)	183
Ns (% genome)	1.5
Annotation statistics	
# gene loci	28,138
Mean gene loci length (bp)	2096.35
Mean CDS size (bp)	1106.32
Median CDS size (bp)	1021
Mean exon size (bp)	242.90
Median exon size (bp)	135
Mean # exons/transcript	5.30
Median # exons/transcript	4

MAKER and Augustus gene prediction generated a final set of 28,138 gene models with an average length of 2096 bp (Table 3). For comparison, the most recent annotation of the *A. thaliana* genome (Araport11) has 27,655 protein‐coding gene models. Over 99% of the gene set was annotated from protein databases with the remainder from the RNA‐seq. More than 99% of the annotated genes possessed an AED score < 0.5 suggesting our reference genome is of good annotation quality based on the general benchmark figures of Campbell et al. (2014). Functional annotations could be assigned to 75% of the genes.

Overall, 53.86% of the genome was indicated as repeat sequences (Table S6), with retroelements making up 15.5 MB (8.63%) of the genome with Gypsy (4.62% of the genome) and Ty1/Copia (1.98% of the genome) the most abundant repeat element families. DNA transposons comprise 1.27%of the genome. A total of 50.7 Mb (28.21%) of the assembled genome was masked by RepeatMasker (interspersed repeats, small RNA, satellites and low complexity).

### 3.2 Comparative phylogenetics, divergence estimates and gene family expansions and contractions

A total of 33,288 orthogroups were recovered across the 13 proteomes, with 11,103 orthogroups shared between all 13 Brassicaceae species. For *D. millefolia*, 97.4% of gene models were assigned to orthogroups, with 728 unassigned genes (2.6%) grouped into 96 orthogroups specific to *D. millefolia*. *Descurainia millefolia* shared the largest number of orthogroups with *D. sophioides* (18,725), closely followed by *Arabidopsis thaliana* (18,077).

**FIGURE 3:**
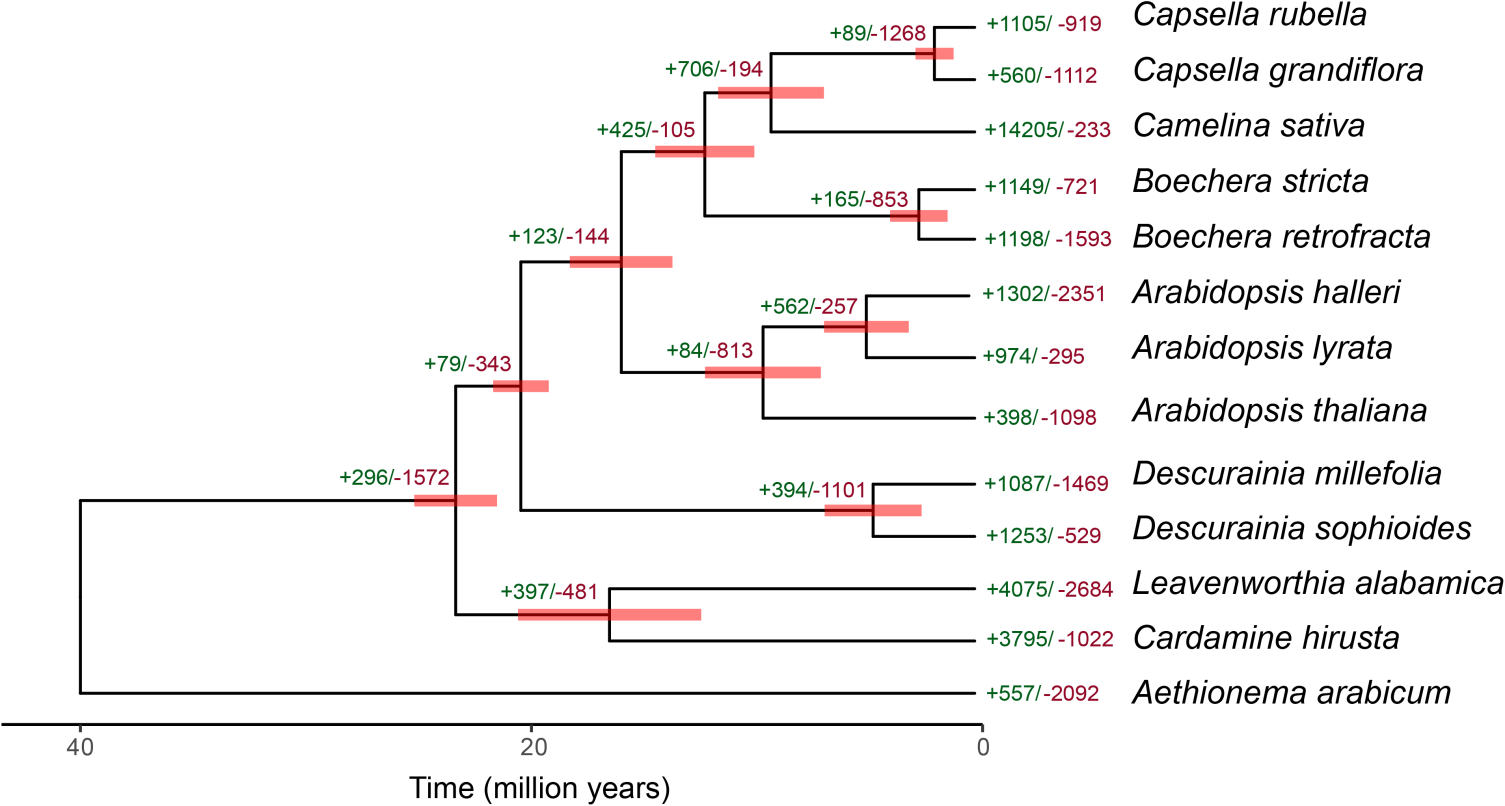
Species tree generated from 2563 single‐copy orthogroups for 13 species within the Brassicaceae, with *Aethionema arabicum* as the outgroup. The red bar on the nodes represents divergence estimates (million years), with their 95% confidence intervals, from PAML MCMC. Gene family expansions (green) and contractions (red) generated by CAFÉ5 are provided on the nodes and species tips.

The species tree (Figure 3), generated from a concatenated alignment of 2563 single‐copy orthogroups where a minimum of 92.9% of species contain single‐copy genes in each orthogroup, consisted of five clades that form successive sister groups: (1) *Leavenworthia* and *Cardamine*, (2) *Descurainia*, (3) *Arabidopsis*, (4) *Camelina* and *Capsella* and (5) *Boechera*. The clade comprising (1) to (5) is estimated to have diverged from *Aethionema arabicum* 39.7 Mya (95% CI: 32.8–45), and the split for the two major clades (i.e. clade 1 vs. clades 2–5) occurred 23.0 Mya (95% CI: 21.41–25.8). *Descurainia millefolia* and *Descurainia sophioides* diverged ca. 4.5 Mya (95% CI: 2.63–6.92).

Comparing *D. millefolia* to *D. sophioides*, that is, the branch that differentiates the Canary Island taxon from a continental taxon, reveals an expansion of 1087 gene families and a contraction of 1469 (*p* < .05) in the former. Among these there were three significant (FDR < 0.05) KEGG pathways each for the contracted and expanded gene families in *D. millefolia* (Table 4). On the branch leading to *Descurainia* there was an increase and decrease in the size of 394 and 1101 gene families respectively.

**TABLE 4:** Significantly overrepresented KEGG functional annotations for the expanded and contracted gene families in *Descurainia millefolia*.

Gene families	Pathway	# Genes	Pathway genes	Fold enrichment
Contracted	Circadian rhythm	7	39	5.34
	Spliceosome	18	187	2.86
	Ribosome	29	315	2.74
Expanded	ABC transporters	6	32	6.61
	Aminoacyl‐tRNA biosynthesis	7	57	4.33
	Biosynthesis of secondary metabolites	65	1243	1.84

### 3.3 Resequencing and selection analysis

#### 3.3.1 Whole‐genome resequencing, mapping, SNP calling and filtering

After trimming, ca. 95% of reads were retained except for one sample where only 73% were retained, resulting in 33.4–49.5 M reads retained per sample (Table S7). Over 95% of the reads from the Canary Islands samples and 71% of the outgroup reads mapped to the reference genome, giving a per sample read depth of >30× and coverage of >99% (Table S7). A total of 1,483,762 variable sites were identified, and after filtering, a dataset of 353,296 high‐quality SNPs was retained for further analysis.

#### 3.3.2 Species relationships

**FIGURE 4:**
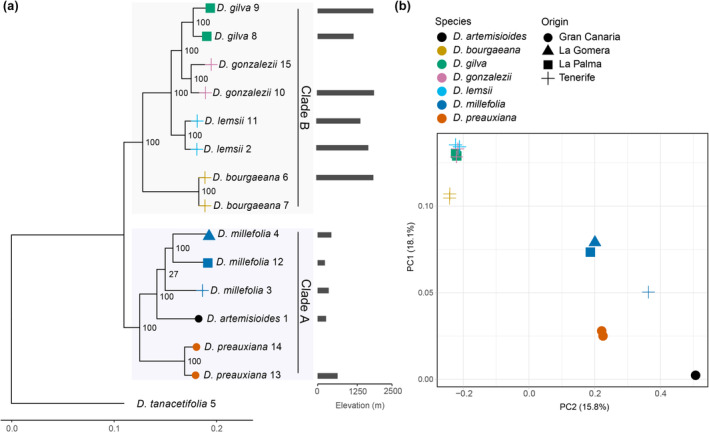
Relationships among the Canary Island *Descurainia* taxa. (a) Maximum likelihood phylogeny using 350 k SNPs from 14 individuals of Canary Island *Descurainia* rooted with *D. tanacetifolia* from the Mediterranean. Species ID numbers can be found in Table 1, tip colours represent island of origin, numbers on nodes represent bootstrap values. (b) Principal component analysis (PCA) representing PC1 and PC2 of the same data. Colours and shapes represent species and island of origin respectively.

The maximum likelihood phylogenetic reconstruction using our SNP datasets provided a well‐resolved phylogeny (only one node shows BS < 100%) and resolved two main clades with maximal support (Figure 4a). This matches the previous phylogenetic analyses (Jackson et al., 2023) and supports the separation of low elevation (*D. preauxiana*, *D. millefolia* and *D. artemisioides*) and high elevation (*D. gilva*, *D. gonzalezii*, *D. lemsii* and *D. bourgaeana*) species. Individuals are always grouped within species, however the Tenerife population of *D. millefolia* is poorly supported as sister to the other *D. millefolia* samples. The PCA provides a similar result, where individuals within the two clades cluster (Figure 4b) and there is a clear separation between the two groups. All individuals within the species cluster tightly except for Tenerife *D. millefolia*, which again is distinct from its conspecifics sampled from La Gomera and La Palma. Three taxa within *D. millefolia* have been described: *D. millefolia* var. *brachycarpa* Bornm. ex Pitard & Proust. (Pitard & Proust, 1908) from Western La Palma, *D. millefolia* var. *sabinalis* O.E. Schulz (Schulz, 1924) from Tenerife, and *D. millefolia* var. *macrocarpa* Pitard. from La Gomera, Tenerife and La Palma (Pitard & Proust, 1908). However, due challenges in delimiting their characteristics and inconsistencies in morphology under cultivation (Bramwell, 1977), more detailed sampling and molecular studies are needed to provide a more robust re‐assessment of the infraspecific taxonomy of *D. millefolia*.

#### 3.3.3 Identification of genes under selection

**FIGURE 5:**
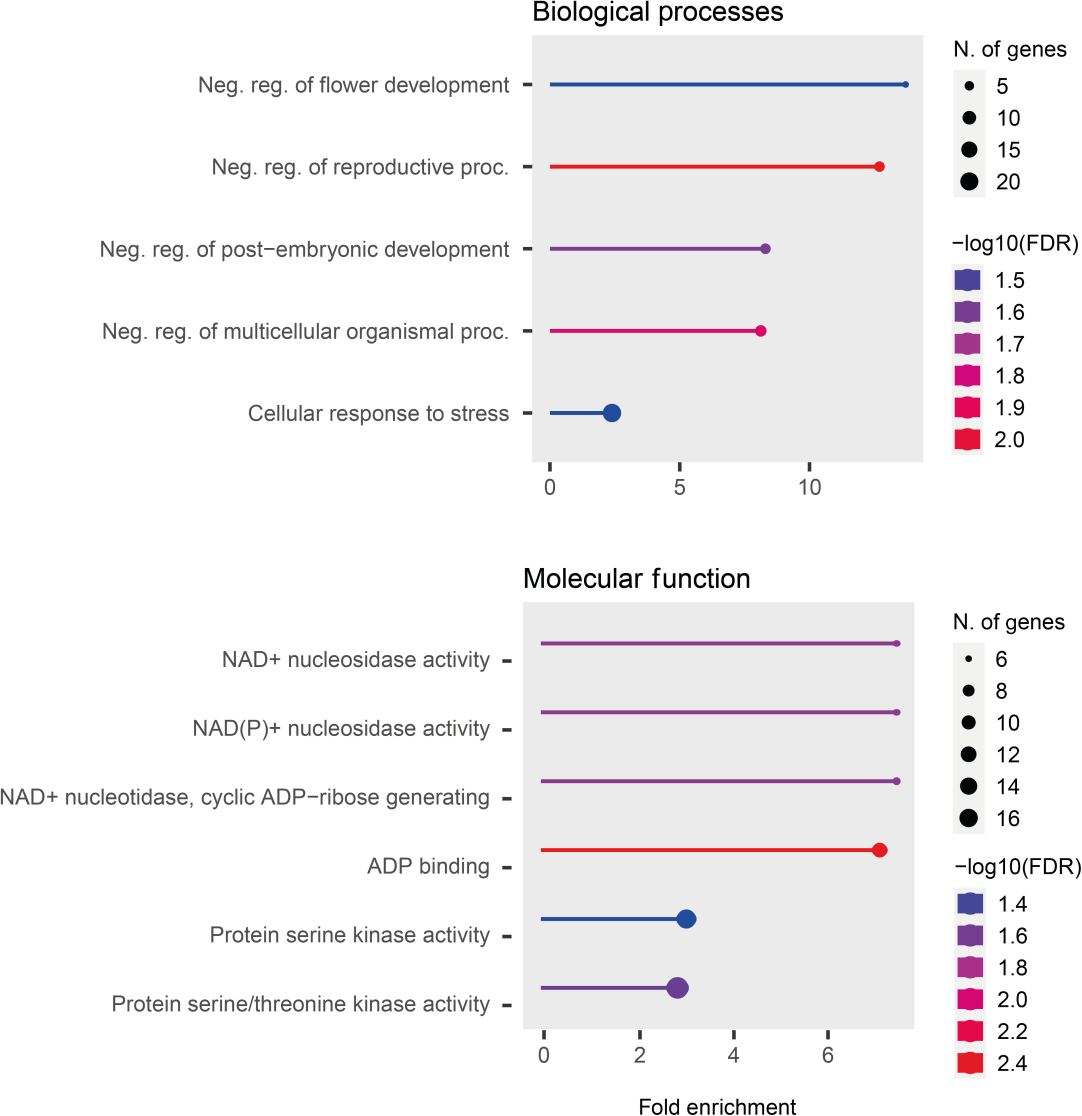
Significantly enriched biological processes and molecular function GO terms (FDR < 0.05) for the PSGs found on the branch separating the low‐ and high‐elevation clades of Canary Island *Descurainia*, ranked by fold enrichment. The size of the dot represents the number of genes. Colour represents FDR corrected *p*‐value (log10) and the *X*‐axis is the level of fold enrichment. Fold enrichment is defined as the percentage of genes belonging to a pathway, divided by the corresponding percentage in the background gene set.

We filtered the set of genes to only include those where all species were present in the alignment, resulting in 25,394 genes (90% of the total genes in the *D. millefolia* reference). From this, we identified 275 positively selected genes (PSGs; ca. 1.1% of the total genes examined) on the branch separating the low‐ and high‐elevation clades (Table S8). After excluding 35 genes (12.7%) of unknown function, GO enrichment analysis found five significantly enriched BPs and six enriched MF terms (FDR‐corrected *p* < .05; Figure 5; Table S9).

Four of the enriched BPs are clearly related and describe the regulation of various flowering and early developmental processes and therefore share many of the same genes; notably, *MAF1* and *MAF2/AGL31* (two *MADS AFFECTING FLOWERING* genes), *APUM9* (*PUMILIO 9*), *FRI* (*FLOWERING LOCUS A*) and *MET1* (*DECREASED DNA METHYLATION 2*). Two of these GO terms also share the gene *CRSP* (*CO*_*2*_ *RESPONSE SECRETED PROTEASE*). The fifth pathway is *cellular response to stress*, which comprises 21 PSGs of which 20 have a putative *Arabidopsis* orthologue (Table S9). Four significantly enriched MF pathways (FDR < 0.05) were related to Nicotinamide adenine dinucleotide (NAD) or NAD(P) and share several genes (Table S9). The remaining two MF terms are related to *protein serine kinase activity* and several genes are shared between these two terms.

### 3.4 Repeated evolution across the Brassicaceae

**FIGURE 6:**
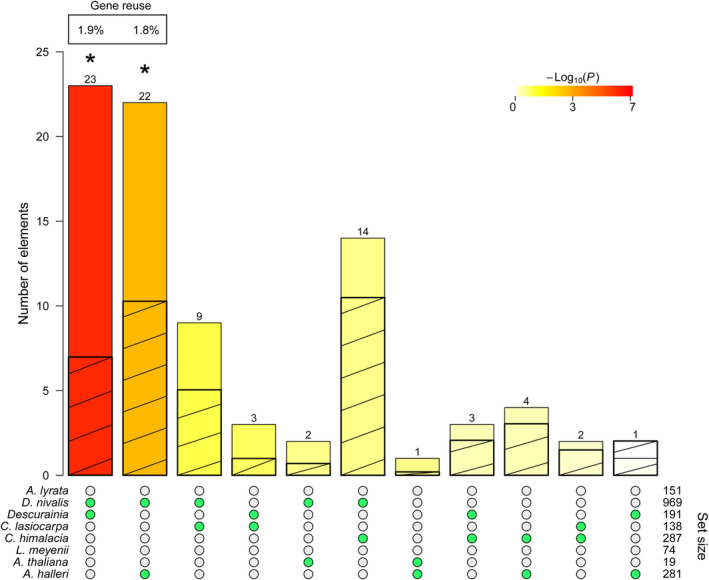
Pairwise overlap in PSGS between studies into high‐elevation or high‐latitude adaptation in the Brassicaceae (ranked by *p* value; see scale). Green dots show species pairs in each case. Bars represent the observed overlap, with and hatched bars representing expected overlap. Significant (*p* value < .05 after FDR correction) intersections are indicated by asterisks and the percentage (%) of gene reuse is shown where the intersection is significant.

Using *Arabidopsis* annotations for sets of PSGs from seven studies (Table S10; and our own from *Descurainia*), 256 multiple comparisons were carried out to examine overlap. No overlap of genes was revealed when more than two studies were considered. Among the pairwise tests, 11 had at least one gene which overlapped, and two pairs showed significant overlap (Bonferroni‐adjusted *p* < .05, Fisher's exact test; Figure 6; Table S11). The comparison of PSGs in *Descurainia* and Arctic‐alpine *Draba nivalis* revealed 23 overlapping PSGs and gave a gene reuse probability of 1.98%. Sixteen of these genes have GO terms associated with them, including *response to cold* and *root development* (Table S12). The second significant overlap was between *Arabidopsis halleri* and *D. nivalis*, where 22 genes overlapped (gene reuse probability of 1.76%). Seventeen of these genes have GO terms associated including *flowering development*, *hyperosmotic salinity response* and *stomatal closure*.

## 4 DISCUSSION

The diversification of plant lineages in the Canary Islands is strongly influenced by the climatic cline exhibited on the islands and particularly the taller islands (Tenerife and La Palma) and differences in climate and elevation ranges correlate with the separation of Canary Island *Descurainia* into low‐ and high‐elevation clades (Jackson et al., 2023). Using 14 genome representing the seven endemic species of Canary Island *Descurainia*, we identified 275 genes putatively related to low‐ versus high‐elevation adaptation. When comparing positively selected genes in Canary Island *Descurainia* with those identified in other species in Brassicaceae that occupy similar environments, we found little evidence of convergence between species, indicating substantially different molecular trajectories to adaptation.

### 4.1 The *Descurainia millefolia* genome

The final assembly of the *D. millefolia* genome is of high quality with an N50 of 0.29 Mb. While it demonstrates a level of fragmentation, the BUSCO results (97.6%) suggest high completeness. The number of gene loci models predicted is 28,138, comparable to *A. thaliana* (27,416) and *Capsella rubella* (26,521) (Slotte et al., 2013; The Arabidopsis Genome Initiative, 2000) and slightly fewer than estimated for *D. sophioides* (30,141 gene models; Chen et al., 2022). Therefore, the final assembly is likely to provide sufficient resources for further downstream genomic analysis.

A total of 25.7% of the genome was classed as interspersed repeats with 8.63% of the genome (15.5 Mb) comprise Retroelements. The repeats of most Brassicaceae genomes are dominated by long terminal repeats retrotransposons (LTR‐RTs), and this is also the case for *D. millefolia* with 7.18% of the genome (ca. 12 Mb; Willing et al., 2015). The genome of *Arabidopsis lyrata* exhibits similarity in LTR‐RTs representation to *D. millefolia* compared to other *Arabidopsis* species. Specifically, *A. lyrata* contains 7.4% and 3.5% of Gypsy and Copia LTR‐RT elements, respectively, mirroring similar genome sizes.

We constructed a species tree of Brassicaceae ‘Lineage I' using the *D. millefolia* proteome along with 12 additional proteomes obtained from either the BRAD v.3.0 databases (Chen et al., 2022) or Phytozome V13 (Goodstein et al., 2012). Our analysis revealed *D. millefolia* and *D. sophioides* form a clade sister to the *Arabidopsis* clade and *Boechera*, *Camelina* and *Capsella* clade, consistent with previously published phylogenetic analyses (Al‐Shehbaz et al., 2006; Liu et al., 2021; Nikolov et al., 2019). Phylogenomic analyses estimate that the split between *Descurainia* and the rest of the sampled Brassicaceae in Lineage I occurred 20 Mya (95% CI: 19–21.5), aligning with previous studies (Guo et al., 2021). Notably, the divergence between the two *Descurainia* species was estimated at 4.5 Mya (95% CI: 2.6–6.8 Mya), indicating a more recent split of the island and continental taxa than previously estimated (Goodson et al., 2011).

The colonisation of the Canary Islands by *Descurainia* would require adaptation to new environmental pressures after arrival. Comparing the *D. millefolia* genome with the 12 other Brassicaceae species revealed significant expansion or contraction in the size of several gene families, offering insights into adaptive evolution. In particular, we observed the expansion of the KEGG pathway involving ABC transport genes, crucial for regulating the movement of molecules across the cell membrane (Hollenstein et al., 2007). ABC transporters play pivotal roles in defending against various stressors, including pathogens, soil minerals and environmental challenges (Banasiak & Jasiński, 2022; Kang et al., 2011; Kretzschmar et al., 2011).

### 4.2 Genomic divergence of the genus *Descurainia* in the Canary Islands

Climate can have a significant influence on plant morphological, physiological and life‐history characteristics, for example, in vernalisation, the timing of flowering or bud burst (Alberto et al., 2013; Csilléry et al., 2014; Leinonen et al., 2009; Mishra et al., 2020; Nowak et al., 2021; Zhang et al., 2019). Our previous phylogenetic work using reduced representation sequencing (Jackson et al., 2023) showed that Canary Island *Descurainia* had undergone one climatic shift which we hypothesise to be associated with the differences in elevational range (i.e. Clade A species are found below 1100 m, and Clade B species above ca. 1800 m; Jackson et al., 2023). The locations from which higher‐elevation species were sampled exhibited lower mean temperatures, lower vapour pressure and higher wind speeds year‐round, higher precipitation during the winter and higher solar radiation in the summer. These results are consistent with the typical characterisation of alpine and subalpine habitats (Nagy & Proctor, 1997). In keeping with Jackson et al. (2023), the phylogeny presented here resolves two altitudinally and climatically distinct clades. The only difference between this study and the Jackson et al. (2023) study concerns the position of *D. lemsii* within the high‐elevation clade and this does not impact on the transition between low‐ and high‐elevation habitats that is the focus here.

Using a Branch‐Site selection approach we found a total of 275 genes (1.1% of available genes) showed evidence for significant positive selection on the branch separating the low‐ and high‐elevation species in the phylogeny. Our GO (Gene Ontology) enrichment analysis found over‐representation of pathways potentially related to adaptation to such habitats, such as reproduction and development and abiotic stress.

For example, associated with the branch between the low‐ and high‐elevation taxa, we identified seven PSGs representing four overlapping GO terms associated with reproduction and development, notably negative regulation of flowering development and negative regulation of reproductive process. Of these genes, *FRI* induces late flowering (Fournier‐Level et al., 2022), and the *MADS AFFECTING FLOWERING* (*MAF*) gene family (*MAF1* and *MAF2/AGL31*) regulates flowering time (Ortuño‐Miquel et al., 2019; Ratcliffe et al., 2003; Rosloski et al., 2013; Scortecci et al., 2001). Changes in reproductive strategies are often expected within high‐elevation floras (Cheviron & Brumfield, 2012; Feng et al., 2022; Leinonen et al., 2009). Of the Canary Islands *Descurainia* species, *D. bourgaeana* and *D. gilva* occur at the highest elevations (1800–2300 m) and have a flowering period of 1 month (ca. mid‐May to mid‐June; Appendix Figure S1) while *Descurainia gonzalezii* and *D. lemsii*, which also occur at high elevations, have a slightly longer flowering period (ca. April–July). In contrast, lower elevation species (i.e. *D. millefolia*, *D. artemisioides* and *D. preauxiana*) have flowering periods of between 5 and 8 months (January–September; Bramwell, 1977). At this stage, however, we do not know whether this is a genetic adaptation or a plastic response to the environment.

We also identified 21 PSGs with the GO term *cellular response to stress* assigned. On Tenerife, the mean annual temperature ranges from ca. 20.8°C on the northeast coast (Santa Cruz meteorological station; 37 m) to 9.8°C in Teide (Izaña meteorological station; 2350 m; Fernández‐Palacios, 1992). With a decrease in temperature, the frequency of frost events also increases. Adaption to lower temperatures is vital for the survival of plants at high elevations and it can have several physiological and molecular consequences (Körner, 2016) and several studies have identified temperature as an important selection pressure for altitudinal adaptation in mountain systems (e.g. Guo et al., 2018; Kubota et al., 2015).

Among the PSGs identified, *ATJ49* is a chaperone cofactor‐dependent protein which enables Hsp70 protein binding (Gaudet et al., 2011). Typically, *HSP70* genes are known to play a critical role in high‐temperature stress response in plants, but the over‐expression of HSP70s has also been found to enhance tolerance to other stresses, such as drought, cold and salt stress (Alvim et al., 2001; Masand & Yadav, 2016; Ono et al., 2001; Tang et al., 2016; Zhao et al., 2019). It is notable that Ma et al. (2015) found an increase in the accumulation of HSP70 with increase altitude in the alpine plant *Potentilla saundersiana*. Another gene under this GO term, *CLPB4*, confers thermotolerance to chloroplasts during heat stress (Myouga et al., 2006).

We also identified a gene orthologous to *ERCC1* (*UV hypersensitive 7*), a DNA repair gene which plays a key role in response to UV‐B and gamma radiation (Dubest et al., 2004; Hefner et al., 2003; Jiang et al., 1997). For every 1000 m in elevation, the UV radiation level will increase by about 12% as the thinner atmosphere filters out less UV radiation (Blumthaler et al., 1997). An excess of UV radiation is well known to result in DNA damage, including mutagenic and cytotoxic DNA lesions (Jansen et al., 1998; Sinha & Häder, 2002; Strid et al., 1994; Tevini & Teramura, 1989; Verdaguer et al., 2017). In Hawaii, plant species at higher elevations have been found to be less sensitive to UV‐B radiation (Sullivan et al., 1992).

Water availability and drought have been associated with high‐elevation environments (Nagy & Grabherr, 2009), and drought tolerance has been demonstrated in several Canary Island species (González‐Rodríguez et al., 2017; Grill et al., 2004; Lausi & Nimis, 1986; López et al., 2008, 2009). Several genes putatively associated with water availability, based on GO terms, were found in the PSGs. Another PSG was RD29B, which is unregulated in response to various abiotic stressors, including cold and desiccation (Nakashima et al., 2006; Yamaguchi‐Shinozaki & Shinozaki, 1993).

Adaptation to a novel environment often leads to exposure to new diseases and pathogens. Several genes under the significantly enriched molecular function GO terms (e.g. RLM1B, RLM1A and RPP1) also have annotations related to bacterial and fungal defence (Creff et al., 2010; Rehmany et al., 2005; Staal et al., 2006). There is currently a lack of research focusing on microbial communities across the Canary Islands, but other studies have found associations between soil composition and plant diversification. Most notably, Osborne et al. (2018) demonstrated that plant pathogens (fungal and bacterial) differ between soil types on the oceanic island of Lord Howe and the sympatric speciation of *Howea* palms is associated with soil preference (Savolainen et al., 2006).

### 4.3 Repeated evolution across Brassicaceae species adapted to high‐elevation and latitude environments

Repeated evolution offers valuable insights on the predictability of evolutionary change, particularly in understanding the emergence of traits in response to environmental pressures. It can be expected to occur in similar environments because they exert similar selection pressures, generating non‐random and repeated outcomes in independent lineages (Stuart, 2019). However, with increase divergence between species, genetic parallelism is seen to decrease due to divergence of availability of preexisting or ancient genetic variation (Bohutínská et al., 2021). Despite increasing evidence of repeated evolution, our understanding of the factors influencing its likelihood and the extent of genomic parallelism within Brassicaceae remains limited.

When comparing our list of positively selected genes separating low‐ and high‐elevation species of Canary Island *Descurainia* with those resolved in studies of adaptation in Brassicaceae species that occupy high‐elevation or high‐latitude environments, we found little evidence of repeated evolution. However, there was a significant overlap between the PSGs in *Descurainia* and those in *Draba nivalis* (Nowak et al., 2021) and the overlapping PSGs are associated with abiotic stressors commonly related to cold and dry conditions seen for both alpine environments for *Descurainia* and arctic conditions for *D. nivalis*, suggesting similar molecular trajectories to adaptation. Of the 23 PSGs shared between *Descurainia* and *Draba*, at least three (13%) have been associated with abiotic stressors. One such gene is *OSCA1*, an osmosensor responsible for Ca2+ increase induced by osmotic stress in plants (Pei et al., 2022; Zhang et al., 2020). In addition, the *LOX1* gene has been shown to be induced during root responses to osmotic stress (Chen et al., 2021), and transgenic mutants have been shown to increase the length of the primary root (Vellosillo et al., 2007). It is possible that LOX1 is involved in deeper root formation during a period of water stress, a common adaptation found within alpine plants (Bliss, 1962). Finally, the gene PME41 is an important cell wall enzyme that plays a role in cold and freezing tolerance (Qu et al., 2011). Overlap was also resolved for the *D. nivalis* and *Arabidopsis halleri* studies. Our results align with those of Birkeland et al. (2020) who showed limited overlap in the genes involved in cold adaptation in arctic Brassicaceae, but a degree of overlap in the pathways in which these genes function.

## 5 CONCLUSION

As whole‐genome sequencing becomes more economically viable, more plant genomes within the Brassicaceae are being generated. High‐quality genomes create avenues for the continued development of our understanding of evolution and divergence. Here, we present the first high‐quality scaffold‐level *D. millefolia* genome. There is potential for *Descurainia* to further our understanding of adaptation since within the Canary Island *Descurainia* clade, species occupy habitats with distinct environmental and climatic conditions suggesting a role for adaptation in the diversification of the genus. Recently, there has been an explosion in research on species found in extreme environments and high elevation, utilising genome sequencing to understand adaptation at a molecular level (Guo et al., 2018; Hu et al., 2021; Nowak et al., 2021).

The *D. millefolia* reference genome provides a foundation for advancing hypothesises on the evolutionary processes involved in the diversification of the group. Through comparison of the high‐elevation versus low‐elevation species using whole‐genome resequencing, we identified several genes associated with environmental stressors that likely play a role in determining the trajectory of evolution for species colonising high‐elevation habitats. Understanding the genomic basis of adaptation to extreme stressors within wild plants is still within its infancy but can ultimately further our understanding of the evolutionary mechanisms behind how organisms thrive in some of the most inhospitable environments.

## AUTHOR CONTRIBUTIONS

**Amy C. Jackson:** Formal analysis (lead); investigation (equal); writing – original draft (lead). **Mark A. Carine:** Conceptualization (equal); funding acquisition (equal); investigation (equal); supervision (equal); writing – review and editing (equal). **Mark A. Chapman:** Conceptualization (equal); funding acquisition (equal); investigation (equal); project administration (lead); supervision (equal); writing – review and editing (equal).

## FUNDING INFORMATION

This work was supported by a PhD studentship to ACJ from the Natural Environmental Research Council (grant number NE/L002531/1).

## CONFLICT OF INTEREST STATEMENT

The authors declare no conflicts of interest.

## BENEFIT SHARING STATEMENT

This manuscript represents a collaboration between MCa and MCh which included co‐supervising ACJ during her PhD and all are co‐authors. All samples were collected using appropriate permits (see methods) and are consistent with the stipulations of the Nagoya Protocol.

## Supporting information


Figure S1.

Table S1.–S12.

## Data Availability

Sequencing data are available through SRA (PRJNA1060497); the genome is available through figshare (https://doi.org/10.6084/m9.figshare.24619074.v1).
