# Supplementary material for: Genomics of ecological adaptation in Canary Island Descurainia (Brassicaceae) and comparisons with other Brassicaceae
Source: Ecol Evol. 2024 Aug 8;14(8):e70144. doi: 10.1002/ece3.70144 (PMC11307170; doi:10.1002/ece3.70144)
Supplement: Supplementary file 1 — Figure S1. [file ECE3-14-e70144-s001.docx]

Appendix Figure S1: Distribution of flowering herbarium specimens (deposited within the Natural History Museum, UK) of the seven Canary Island *Descurainia* species, according to the month of collection.
